# Economic impact of disposable versus reusable instruments in minimally invasive surgery: a systematic review

**DOI:** 10.1007/s00464-026-12765-x

**Published:** 2026-04-22

**Authors:** Pieter J. W. Greve, Masie Rahimi, Freek Daams, Jan Willem M. Greve, Jaap H. Bonjer, Tim Horeman, Marlies P. Schijven

**Affiliations:** 1 Dept. of Surgery, Amsterdam UMC Location, De Boelelaan 1117, Amsterdam, The Netherlands; 2 Amsterdam Gastroenterology and Metabolism, Amsterdam, The Netherlands; 3 Amsterdam Public Health, Digital Health, Amsterdam, The Netherlands; 4 Department of Biomechanical Engineering, Delft University of Technology, Delft, The Netherlands; 5 Maastricht NUTRIM - Institute of Nutrition and Translational Research in Metabolism, Maastricht, The Netherlands; 6 Cancer Center Amsterdam, Amsterdam, The Netherlands; 7 Science Hub for ASC Research and Education (SHARE), Amsterdam Skills Centre for Health Sciences (ASC), Amsterdam, The Netherlands

**Keywords:** Minimally invasive surgery, Surgical instruments, Disposable instruments, Reusable instruments, Cost analysis, Environmental sustainability, Life-cycle assessment

## Abstract

**Objective:**

To systematically evaluate the economic impact of disposable versus reusable instruments in minimally invasive surgery (MIS), and to summarize the limited available evidence on environmental impact.

**Background:**

The increasing use of disposable instruments in MIS has raised concerns regarding healthcare costs and environmental sustainability. While reusable instruments may reduce per-procedure costs and waste, their economic and environmental performance is influenced by procedure type, workflow, and reprocessing requirements. Evidence integrating these factors across surgical specialties remains limited.

**Methods:**

A systematic review was conducted in accordance with PRISMA guidelines. Studies published since 2014 comparing disposable and reusable instruments in MIS were identified using predefined PICOS criteria. Data extraction focused on cost components, including instrument costs, sterilization, operating room time, and total procedural costs. Environmental outcomes were recorded when available.

**Results:**

Nine studies encompassing 4,724 procedures across multiple surgical specialties met inclusion criteria. In general surgery, reusable instruments were consistently associated with lower per-procedure costs, with reported savings ranging from $16 to $388. In selected subspecialties, including gynecology, thoracic surgery, and spinal surgery, disposable instruments were associated with reduced operative time, indirectly lowering total costs in specific settings. Only one included study directly assessed environmental impact, providing limited, low-level evidence that reusable instruments may confer environmental benefit primarily when used repeatedly.

**Conclusion:**

Reusable instruments appear to be associated with lower per-procedure costs in general surgery, while disposable instruments may offer context-specific economic advantages in selected subspecialties. Conclusions regarding environmental impact are limited by the scarcity of primary data. Future studies incorporating standardized cost definitions and robust environmental assessments, including life-cycle analyses, are needed to support evidence-based and sustainable instrument selection in MIS.

**Graphical Abstract:**

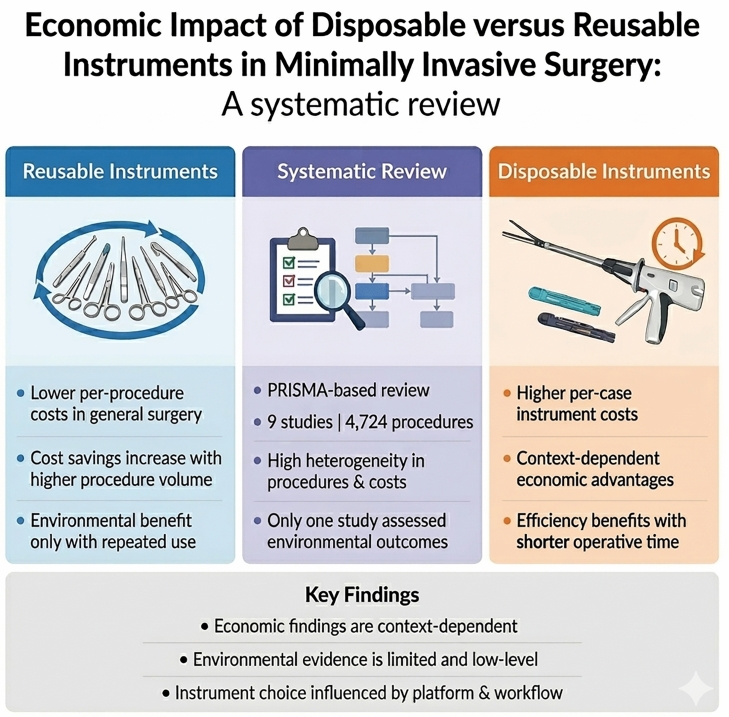

Minimally invasive surgical procedures have revolutionized modern medicine, offering patients reduced recovery times, minimized postoperative pain, and decreased risk of complications compared to traditional open surgeries [1, 2]. With the advancement of these techniques, the use of specialized surgical instruments has become increasingly prominent. These instruments can be broadly categorized into disposable (single use) and reusable (multi-use) items. Both types have distinct advantages and disadvantages, which impact their economic and environmental viability [3, 4].

The cost implication of using disposable versus reusable instruments in minimally invasive surgery is multifaceted. Disposable instruments often have higher per-unit costs, which can accumulate rapidly in high-volume surgical centers [5]. However, they offer advantages such as reduced risk of cross-contamination and lower costs associated with sterilization and reprocessing [6]. Reusable instruments, while incurring higher initial investment and ongoing costs for sterilization, can be more cost effective over time when utilized efficiently [7, 8]. Despite these considerations, there is limited comprehensive synthesis of the existing evidence comparing the overall cost-effectiveness of disposable versus reusable instruments. This review aims to fill this gap by systematically evaluating the economic impacts of these instruments in various surgical settings [9].

The environmental footprint of surgical practice has gained increasing attention in recent years. Disposable surgical instruments contribute significantly to medical waste, which poses challenges for waste management and environmental sustainability [10, 11]. Conversely, reusable instruments, although reducing waste, require resources for cleaning and sterilization, which can contribute to environmental pollution through water use and chemical discharges [12, 13]. The comparative environmental impacts of disposable and reusable instruments are complex and context dependent. There is a pressing need for a systematic review that collates and assesses the environmental data, helping to inform sustainable practices in minimally invasive surgery [14].

Previous studies have individually addressed either the economic or environmental aspects of surgical instruments, but few have integrated both perspectives. Additionally, the available literature often focuses on specific procedures or healthcare settings, limiting the generalizability of findings [15].

This systematic review is designed to provide a comprehensive synthesis of the economic and environmental impacts of using disposable versus reusable instruments in minimally invasive surgical procedures.

## Methodology

### Search strategy

This systematic review was conducted in accordance with the PRISMA (Preferred Reporting Items for Systematic Reviews and Meta-Analysis) guidelines, updated in 2020 [16]. The PICOS criteria [17] were established to align with the study’s objectives (Table 1). All studies published in English that assessed the use of disposable versus reusable components in minimally invasive procedures were retrieved. No filters for specific surgical specialties were applied. All surgeries employing disposable or reusable instruments in robotic, laparoscopic, or endoscopic procedures were included in the primary search.

**Table 1:** PICOS criteria for systematic review [17]

Population	Patients undergoing any minimally invasive surgery
Intervention	Use of disposable instruments/single-use surgical instruments in the minimally invasive procedure under study
Control	Use of reusable/multi-use surgical instruments in the minimally invasive procedure under study
Outcome	Primary: 1. Economic impact of use of disposable vs. reusable instruments in minimal access surgeries 2. Environmental impacts of disposable vs. reusable instruments in terms of waste material production, cost associated with sterilization Secondary: 1. Comparison and cost analysis of disposable and reusable instruments used in two different minimally invasive surgeries 2. Total cost analysis with and without the cost of instruments to highlight the most cost effective minimal invasive surgery
Study design	Randomized controlled trials and non-randomized controlled trials, retrospective/prospective observational studies, case–control studies

### Search string

The search string was developed in collaboration with the Medical Library AMC, Amsterdam UMC. The basic search strategy employed in each database included the following search string: (Minimally Invasive surgery OR Minimal Access Surgery OR Robot-assisted surgery OR Laparoscopic surgery) AND (Disposable OR Multi Use) AND (Reusable OR Single Use) AND (Cost effectiveness) AND (Environmental impact). The search string was translated using the polyglot search [18] to remove the translation bias introduced during the subjective translation. Electronic databases including PubMed, Cochrane Library, Embase, Scopus, and Elsevier were searched, and shortlisted articles were added in the automated reference manager Rayyan [19].

### Inclusion criteria

Studies assessing the cost of disposable versus reusable instruments in minimally invasive surgeries were included in the systematic review if they were published in English after year 2014. Studies eligible for inclusion should account for the total cost of the operation, whether or not disposable instruments are used, as the choice of instruments can influence intraoperative and postoperative outcomes like operation time, bleeding, hospital stay length, revision surgeries, and readmissions, ultimately impacting overall hospital expenses.

Similarly, studies assessing the additional costs associated with sterilization and repacking of reusable devices, as well as costs of waste management for disposable devices, were considered higher level of evidence (Table 2).

**Table 2:** Selection criteria for included studies

Criteria	Inclusion	Exclusion
1. Language	English	All other languages
2. Timeframe of publications	2014– 2024	Published earlier than 2014
3. Study design	Randomized/Non-randomized clinical trial Comparative original articles Case control Retrospective observational studies Prospective observational studies Studies sourced from peer-reviewed journals	Case reports Case series Protocols Reviews Gray literature Retracted Articles
4. Population ethnicity	All	-
5. Target population	Patients undergoing any elective minimally invasive surgery involving use of disposable and reusable instruments	Patients undergoing elective open surgery with use of disposable and reusable instruments Patients undergoing emergency minimally invasive surgeries
6. Outcomes measured/intervention	Studies evaluating the cost-effectiveness of use of single-use vs. multi-use devices in minimally invasive procedure Comparison of cost analysis of 2 different types of minimally invasive surgeries with use of disposable and reusable instruments Studies assessing the environmental impacts of instruments and comparing them to identify the instruments/procedure associated with least waste production	Studies assessing the total cost of minimally invasive procedures without special notice of use of any reusable vs. disposable instruments Studies comparing cost-effectiveness of open vs. robotic surgeries Studies mentioning the model formed to decrease the cost of surgeries with rate of compliance of surgeons Studies doing cost analysis in terms of length of hospital stay, postoperative complications, need of revision surgeries, readmissions, and use of ventilatory supports postoperatively

### Exclusion criteria

Review, meta-analysis, retracted articles, gray literature, case reports published before year 2014, and articles without full-text availability were excluded. Similarly, articles publishing life-cycle assessment models for evaluating the economic and environmental impacts of disposable versus reusable instruments without actual prospective or retrospective data collection were excluded. These studies explain an ideal economic and waste management model based on the instructions given by the manufacturers. However, real-life costs and waste production deviate from these theoretical models. Moreover, surveys assessing the attitude of surgeons toward the burden of costs for instruments opened but not used in surgery were excluded. Although these surveys assess an important cause of increased expenditure per procedure, they do not align with the outcome of the current review, which strictly includes the economic and environmental impacts of disposable versus reusable instruments (Table 2).

### Data extraction

Articles were screened by two individual authors with blinding on. The initially screened and shortlisted articles by both authors were compared, and disputes were resolved by a third author who was not included in the initial screening process to remove selection bias. The decision of the tie breaker was considered final. Full-text articles were reviewed, and shortlisting was done keeping PICOS criteria in mind.

Baseline study characteristics were extracted from the shortlisted articles, including author, year of publication, surgical specialty, clinical condition, minimally invasive techniques used, disposable instruments, reusable instruments and outcomes analyzed (Table 3). Next, a systematic review of the included studies was performed, grouping studies according to their surgical specialty (Table 4).

**Table 3:** Baseline characteristics of the included studies assessing the economic and/or environmental impacts of disposable vs. reusable instruments

Sr no	Author, year	Study design	Country	Sample size	Surgical speciality	Minimally invasive surgery under study	Disposable instrument	Reusable instrument	Outcomes measured
1	Shussman et al.,[21]	Prospective observational cohort study	Israel	34	General surgery (hepatobiliary surgery)	Laparoscopic cholecystectomy	Disposable access device (*n* = 17)	Xcone™ port + reusable pre shaped graspers (*n* = 17)	The cost-effectiveness of the reusable instruments as compared to disposable instruments used previously. Retrospective data collected from hospital registry and propensity-matched latest cholecystectomies performed were used as control
2	Grimes et al, [22]	Comparative observational study	Cleveland, Ohio	65	General surgery (hepatobiliary surgery)	Laparoscopic cholecystectomy	5mm clip applier, disposable cannula, disposable suction, disposable needle for skin closure, Dermabond, and 2 disposable loops per procedure (*n* = 18)	Reusable clip applier, Hasson Cannula, multi-use suction/irrigator, Steri-strips, 1 disposable endoloop per procedure (*n* = 47)	Establishing the main difference in the use of instruments by high cost (> $800/ case) vs. low cost (< $600/case) surgeons and assessing the impact of use of disposable vs. reusable instruments as one of the reasons for the cost difference
3	Abdelmoaty et al. [23]	Comparative observational study	United States of America	2405	General surgery	Elective laparoscopic or robotic inguinal hernia repair	Laparoscopic inguinal hernia repair (*n* = 1671)	Robotic inguinal hernia repair (*n* = 734)	Direct cost associated with the resources directly used for individual surgery was divided into fixed cost, which was excluded while variable cost comprising of disposable vs. reusable instruments was analyzed
4	Hassan et al. [24]	Retrospective observational cohort study	United Arab Emirates	280	General surgery	Elective laparoscopic cholecystectomy	Conventional lap chole (*n* = 140) Disposable 5mm and 10 mm trocars, electrohook, 5mm grasper	Emirate lap chole (*n* = 140) Veress needle, 5 mm port, 1 grasp instrument, electrocoagulation hook, bipolar forceps, Endo clip applicator, 6 polymer clip cartridges	Cost analysis of modified endoscopic limited-access lap chole with reusable ports named as Emirates lap chole and conventional lap chole
5	Cunha et al [25]	Retrospective observational study	Netherlands	298	General surgery (hepatobiliary surgery)	Liver resection using limited-access surgeries	Robotic liver resection using disposable instruments (*n* =)	Laparoscopic liver resection using reusable instruments (*n* =)	Cost Analysis was done as a whole and according to level of difficult operability of the liver lesion. Intraoperative costs including operation theater costs, sterilization, disposable, postoperative costs incl. postop complication, length of stay, readmissions, revision surgeries
6	Han Z. et al. [26]	Retrospective observational cohort	China	1556	Thoracic surgery	Video-assisted thoracoscopic surgery (VATS)	EasyEndo single-use endoscopic cutting and stapling device (n =)	Johnson EC45A staplers (n =)	Intra and postoperative complications and cost-effectiveness of disposable EasyEndo stapling device in comparison to the multi-use control group stapler device
	Holloran-Schwartz MB. et al. [27]	Randomized controlled trial	Saint Louis, Missouri	46	Gynecology and obstetrics	Laparoscopic hysterectomy	Single-use transactor and ligature with 5mm blunt tip with force triad generator (*n* = 23)	Reusable bipolar forceps and reusable monopolar scissors with force triad generator (*n* = 23)	Cost analysis was done with each patient serving as a control group for themselves. Left side of uterus was freed from its ligamentous attachments using the disposable energy generator cautery device, while right side was transacted using multi-use forceps and scissors. The force triad Generator was same in both devices so its cost was excluded. Similarly, cost of reusable scissors was negated since that was the mandatory instrument in the hysterectomy tray
	Bouthors et al. [28]	Prospective observational study	France	40	Orthopedics and spinal surgery	Lumbar arthrodesis	Disposable device group, the sterile ready-to-use SteriSpine™ made of polyacrylamide	Reusable devices the CD Horizon® Legacy™ spinal system and Capstone® cage made of stainless steel	Microcosting analysis done for calculating the processing mean cost of overall procedure for single vs. multi-use devices
	Meissner et al. [29]	Product material analysis	Austria	–	Bariatric surgery, thoracic surgery	Lap sleeve gastrectomy, lap gastric bypass, video-assisted thoracoscopic (VATS) lobectomy	Ethicon’s single-use stapler	Medtronic’s multi-use stapler	Environmental impact analysis in each procedure with disposable and reusable devices done using the number of times the device can be reused according to the manufacturer’s manual. Total waste and total material requirement was assessed for 3 different minimally invasive procedures

**Table 4:** Cost analysis of disposable vs. reusable Instruments in minimally invasive surgeries

Sr no	Author, year	Minimally invasive surgery under study	Disposable instrument	Reusable instrument	Results	Instrument favored	Level of evidence grades of recommendation
1	Shussman et al. [21]	Laparoscopic cholecystectomy	Disposable access device (*n* = 17)	XconeTM port + reusable pre-shaped graspers (*n* = 17)	Significant difference was found between the operative time in min and length of postop stay per days, with disposable instruments having more time and LOS as compared to reusable ports (0.0001, 0.001, respectively). The OT cost was considered fixed cost, the variable costs comprising cost of instruments	Reusable device saves $388 and $240 as compared to disposable single-port and multiport devices, respectively	Level 2b: Cohort study Moderate quality evidence Grade B
2	Grimes et al. [22]	Laparoscopic cholecystectomy	5mm clip applier, disposable cannula, disposable suction, disposable needle for skin closure, Dermabond, and 2 disposable loops per procedure (*n* = 18)	Reusable clip applier, Hasson Cannula, multi-use suction/ irrigator, Steri-strips, 1 disposable endoloop per procedure (*n* = 47)	Disposable instruments accounts for the increasing cost of lap chole when compared with the cost of their reusable counterparts	Reusable devices have shown to increase the potential cost savings from $16 to $276 making one of the most common surgeries more cost effective	Level 2b: Cohort study Moderate quality evidence Grade B
3	Abdelmoaty et al. [23]	Elective laparoscopic or robotic inguinal hernia repair	Laparoscopic inguinal hernia repair (*n* = 1671)	Robotic inguinal hernia repair (*n* = 734)	Average total cost of robot assisted hernia repair was significantly higher than lap repair (*p* < 0.001) incl the increased fixed cost (< 0.001). However, variable cost was significantly more in lap group (< 0.001). Total average cost of Robot assisted was more than lap ($5484 vs. $3235), while variable cost of instruments used was more with lap ($1086 vs. $922)	Lap hernia repair had more variable cost accounting for more disposable items used as compared to robot-assisted repair	Level 2b: Cohort study Moderate quality evidence Grade B
4	Hassan et al. [24]	Elective laparoscopic cholecystectomy	Conventional lap chole (*n* = 140) Disposable 5mm and 10 mm trocars, electrohook, 5mm grasper	Emirate lap chole (*n* = 140) Veress needle, 5 mm port, 1 grasp instrument, electrocoagulation hook, bipolar forceps, endo clip applicator, 6 polymer clip cartridges	Overall cost was lower in Emirates lap chole ($528) as compared to conventional lap chole ($793) using the disposable instruments (*p* = 0.0001)	Emirates lap chole using reusable instruments	Level 2c: Retrospective outcome analysis Moderate quality evidence Grade C
5	Cunha et al. [25]	Liver resection using limited-access surgeries	Robotic liver resection (RLR) using disposable instruments (*n* = 143)	Laparoscopic liver resection (LRL) using reusable instruments (*n* = 155)	RLR has significantly higher disposable-related procedure cost: €2140 as compared to reusable LRL cost: €1477 with a statistically significant difference of € 663	Reusable instruments are more cost effective than disposable instruments	Level 2c: Retrospective outcome analysis Moderate quality evidence Grade C
6	Han Z. et al.[26]	Video-assisted thoracoscopic surgery (VATS)	EasyEndo single-use endoscopic cutting and stapling device (*n* = 781)	Johnson EC45A staplers (*n* = 775)	No significant difference was observed in the intra and postoperative complications was found. This made clear that the increase in cost per procedure is directly linked to the type of instruments used. Results of this analysis showed a significant lower cost with the use of single-use EasyEndo stapler as compared to reusable stapler ($1631.23 vs. $2356.22)	Disposable stapler was more cost effective in VATS lobectomy/segmentectomy in lung carcinoma	Level 2c: Retrospective outcome analysis Moderate quality evidence Grade C
7	Holloran-Schwartz et al. [27]	Laparoscopic hysterectomy	Single-use transactor and ligature with 5mm blunt tip with force triad generator (*n* = 23)	Reusable bipolar forceps and reusable monopolar scissors with force triad generator (*n* = 23)	The cost of single-use device was no doubt more than reusable devices used for lap hysterectomy ($630 vs. $12) However, the use of disposable device decreased the operation time and hence indirectly reduced the overall cost of the procedure ($254.16/case was saved)	Disposable energy devices improve the overall cost of procedure despite being expensive	Level 1b: Randomized controlled trial High-quality evidence Grade A
8	Bouthors et al. [28]	Lumbar arthrodesis	Disposable device group, the sterile ready-to-use SteriSpine™ made of polyacrylamide	Reusable devices the CD Horizon® Legacy™ spinal system and Capstone® cage made of stainless steel	Microcosting analysis done for the reusable spinal surgical set (58.30 €) vs. Single-use spinal set (14.19 €) showed a significant increased cost with reusable instruments due to increased processing time (that is time taken from opening the set till it is either disposed of or resterilized and packed for reuse). However, if sterilization cost is taken into account the reusable instruments become much more expensive since an additional 137.25 € is required to disinfect instruments for each procedure	Owing to disposable nature of the single-use spinal sets sterilization cost is saved making it more cost effective	Level 2b: Cohort study Moderate quality evidence Grade B
9	Meissner et al. [29]	Lap sleeve gastrectomy, lap gastric bypass, video-assisted thoracoscopic (VATS) lobectomy	Ethicon’s single-use stapler	Medtronic’s multi-use stapler	Conversion of single-use staplers to multi-use staplers in lap procedures is considered to significantly decrease the total waste produced and total material required only if the reusable stapler is at least used for four procedures or more. Use of reusable device less than that will make comparable waste to single-use devices	Environmental impact of reusable device is beneficial only if they are used in four or more procedures	Level 5: Mechanistic study Very low-quality evidence Grade D

### Quality assessment

Since a range of study designs were included in the systematic review, level of clinical evidence was assessed using Grades of Recommendation explained by Stony Brook University [20]. The classification of clinical evidence is typically organized into a hierarchy that ranks the strength or quality of evidence based on the study design, reliability, and relevance to clinical practice. The most widely recognized framework for this is the Levels of Evidence, with Level 1 being the high-quality evidence including systematic reviews and meta-analysis conducted on RCTs and Level 5 being very low-quality evidence and comprises expert opinion and mechanism-based reasoning. These levels of evidence can guide decision-making in medicine, particularly for developing clinical guidelines.

## Results

### PRISMA flowchart

**Fig. 1:**
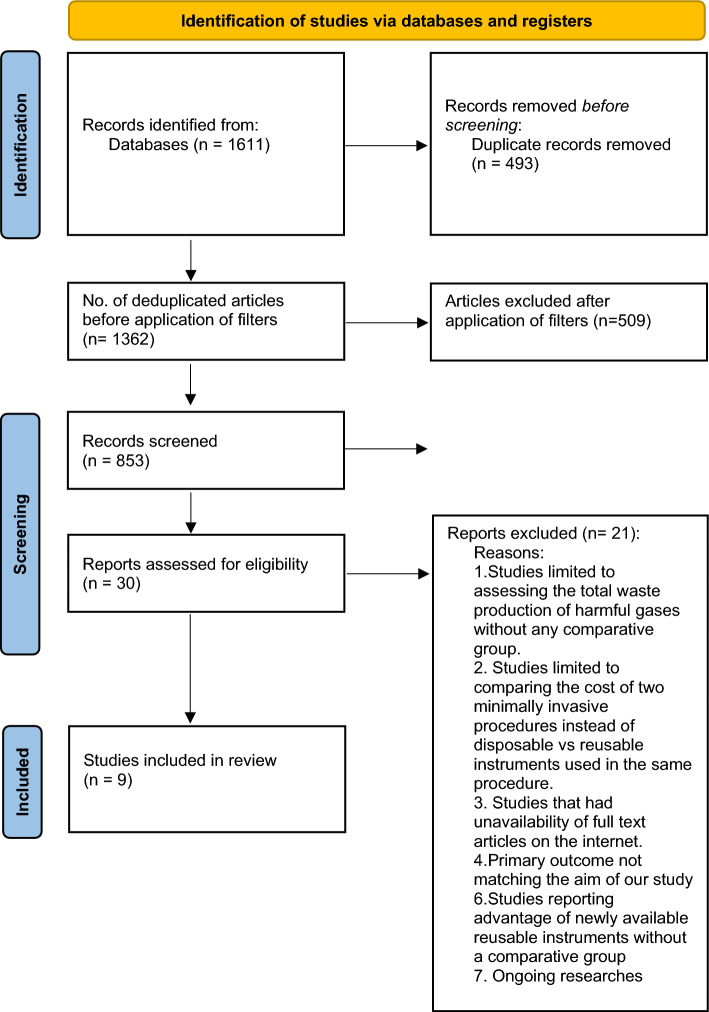
PRISMA flowchart

This systematic review, conducted in accordance with the PRISMA flowchart, followed the screening steps as explained in 2020 checklist [16]. A total of 1611 records were shortlisted from the electronic databases mentioned above. 493 duplicates were resolved after adding the articles to Rayyan. The deduplicated articles were screened according to the PICO criteria, and 853 articles were considered eligible for abstract screening. This revealed a total of 30 articles suitable for full-text review. Finally, 9 full-text articles [21–29] were included in the systematic review (Fig. 1).

### Baseline characteristics

Out of these nine articles, only one assessed the environmental impact of disposable versus reusable instruments in different minimally invasive surgeries. Most studies assessing the burden of waste production associated with disposable instruments were Life Cycle Assessment [30] models, formulating hypotheses in ideal conditions without actual calculation of the waste produced. These were excluded from the final review. A total of 4724 cases were analyzed to assess the economic impacts of disposable (single use) versus reusable (multi-use) devices in minimally invasive surgeries across various surgical subspecialities. Most of the studies (*n* = 5; 55.56%) [21–25] assessed the economic impact of disposable instruments in general surgery procedures, including laparoscopic cholecystectomy (n = 3; 60%) [21, 22, 24], laparoscopic inguinal hernia repair (n = 1; 20%) [23], and limited-access liver resection (*n* = 1; 20%) [25]. Other studies evaluated the cost-effectiveness of different types of instruments in video-assisted thoracoscopic surgery (VATS) [26], laparoscopic hysterectomy [27], and lumbar arthrodesis [28].

### Quality assessment

Among the included studies, one was a randomized controlled trial [27], four were prospective studies [21–23, 28], three were retrospective observational cohort studies [24–26], and one was a product material analysis [29]. Due to the varied study designs, the level of evidence ranged from Level 1b for the RCT, Level 2b for observational cohorts, and Level 5 for the product material analysis. This indicates that most included studies (*n* = 6; 75%) provide moderate-quality evidence.

### Economic impact of disposable vs. reusables in the general surgery department

Systematic reporting of the included studies indicated no significant differences in intraoperative or postoperative complications in laparoscopic cholecystectomy when using either type of instrument. However, using reusable devices significantly decreased the operative cost of laparoscopic cholecystectomy, saving $16 to $388 per case, depending on the disposable counterparts used [21, 22].

Similarly, a study assessing the cost-effectiveness of limited-access inguinal hernia repair with laparoscopy and robotic surgery showed that, despite the high fixed cost of robotic surgery, the variable cost of laparoscopic surgery was significantly higher ($1086 vs. $922) [23]. This increase was due to the use of disposable instruments in laparoscopic repair compared to reusable devices in robotic surgery. Alternatively, liver resection performed laparoscopically was cheaper than those performed robotically. In this procedure, surgeons used more reusable devices during laparoscopic excision, significantly decreasing the overall cost of the procedure from €2140 for robotic surgery to €1477 [25]. These findings suggest that using reusable devices in general surgery significantly reduces the variable cost of instruments per procedure, making them more cost effective and affordable.

### Economic impact of single-use vs. multi-use devices in surgical subspecialties

A cohort study assessing the cost-effectiveness of disposable vs. reusable stapler showed that cost per procedure was significantly reduced when disposable stapler was used for closing gaps after lobectomy or segmentectomy in lung carcinoma [26]. Similarly, the use of disposable spinal sets for lumbar arthrodesis was found to be more cost effective in a 2019 cohort study conducted by Bouthors et al. on 40 cases [28]. This cost-effectiveness of disposable devices in comparison to reusable sets was due to increased sterilization cost (€137.25) required for processing reusable instruments.

Additionally, Holloran-Schwartz et al. (2015) conducted a randomized controlled trial on 46 patients undergoing laparoscopic hysterectomy to analyze the costs of disposable vs. reusable energy generators used for transection of uterine ligamentous attachments [27]. In this study, one side of the patient’s uterus served as a control and was dissected using a disposable blunt-tip transactor, while the other side was separated using reusable mono- and bipolar forceps. Results showed that while the cost of disposable device was higher than the reusable devices ($630 vs. $12), its use reduced operation time, thereby indirectly lowering the overall cost of the procedure. A total of $254.16 per case was saved.

### Environmental impact of disposable vs reusables in minimal access surgeries

Finally, only one study analyzed the environmental impact of disposables in terms of total waste production and total material requirement after dissembling the instruments into their basic components [29]. This study found that the shift from disposable staplers to reusable staplers in VATS, laparoscopic sleeve gastrectomy, and laparoscopic gastric bypass is only beneficial if the reusable instrument is used in more than four procedures. Using a reusable device fewer than four times generates waste comparable to that of disposable devices. Although the level of evidence for this study was very low, it highlights the environmental impact of disposables vs. reusables in addition to their cost-effectiveness.

## Discussion

This systematic review evaluated the economic and environmental implications of disposable versus reusable instruments used in minimally invasive surgical (MIS) procedures. The interpretation of cost-effectiveness in this review is limited by variability in cost definitions, perspectives (hospital vs. procedural), and inclusion of fixed versus variable costs across studies.

The principal finding is that reusable instruments are generally associated with lower direct procedural costs in general surgery, whereas disposable instruments may confer conditional economic advantages in selected subspecialties, primarily through reductions in operative time [21–25, 27, 28]. However, the environmental evidence base remains limited, precluding robust conclusions regarding sustainability [29, 31, 32].

Across the included studies, reusable instruments were consistently associated with reduced per-procedure costs in general surgical procedures, most notably laparoscopic cholecystectomy [21, 22, 24]. These cost savings were primarily driven by lower instrument acquisition costs when amortized over repeated use [21, 22]. In contrast, in subspecialties such as gynecology, thoracic surgery, and spinal surgery, disposable instruments were sometimes associated with lower total costs, largely attributable to shorter operative times or reduced reprocessing costs rather than lower device costs per se [26–28].

Importantly, the definition of “total cost” varied substantially across studies. Some analyses focused narrowly on instrument acquisition costs [21, 22], whereas others incorporated operating room time, sterilization, maintenance, or downstream costs [25–28]. Moreover, few studies explicitly distinguished between fixed and variable costs, limiting cross-study comparability [23, 25]. As a result, the economic findings should be interpreted as context dependent rather than universally generalizable. Instrument-related costs are inseparable from institutional workflows, surgeon experience, contractual pricing, and health system reimbursement structures [23, 28, 33].

A key limitation identified in this review is the frequent confounding of instrument type with surgical platform. Several studies compared disposable instruments used in robotic surgery with reusable instruments used in conventional laparoscopy, making it difficult to isolate the independent economic effect of disposability [23, 25]. Instrument choice is often embedded within broader technological ecosystems, including robotic platforms, standardized procedural kits, and institutional purchasing agreements. Consequently, cost differences observed across studies likely reflect system-level factors rather than instrument characteristics alone [23, 25, 31].

The use of disposable versus reusable instruments in MIS also carries important environmental implications. Disposable instruments, while reducing the risk of cross-contamination and improving convenience, contribute to increased material consumption and medical waste generation, raising concerns regarding environmental sustainability [31, 34–36].

Although environmental sustainability was a stated objective of this review, only one included study directly assessed environmental outcomes, and this study provided low-level evidence [29]. The absence of comprehensive life-cycle assessment (LCA) data—including manufacturing, reprocessing, transportation, and end-of-life disposal—severely limits the strength of environmental conclusions [30–32, 34]. While the available evidence suggests that reusable instruments may offer environmental advantages when used above a minimum reuse threshold, this finding remains hypothesis-generating rather than definitive [29].

While not included in the systematic review due to methodological differences, a 2022 life-cycle assessment by Boberg et al. evaluating disposable and reusable trocars in laparoscopic cholecystectomy demonstrated substantially higher environmental impact for disposable trocars, including greater climate impact and reduced ecosystem quality, strongly favoring reusable devices. Life-cycle assessment studies without direct clinical cost or utilization data were excluded from the systematic review but are discussed narratively where relevant to contextualize environmental considerations [37]. These findings provide contextual support for potential environmental advantages of reusable instruments but do not alter the limited strength of evidence identified in the present review.

Given the growing emphasis on environmentally sustainable surgical practice, the paucity of high-quality environmental data represents a critical knowledge gap. Future research should prioritize standardized LCA methodologies alongside economic evaluations to enable balanced, evidence-based decision-making [31, 32, 34].

From a clinical and institutional perspective, these findings suggest that reusable instruments may be economically favorable in high-volume general surgical settings, whereas disposable instruments may offer efficiency-related benefits in selected contexts [21–25, 27, 28]. However, decisions regarding instrument procurement should account for local case mix, surgical volume, platform integration, and institutional cost structures rather than relying on generalized conclusions [23, 28, 33].

## Conclusion

Reusable instruments appear to be associated with lower per-procedure costs in general surgery settings studied, while disposable instruments may offer context-specific economic advantages related to operative efficiency in certain subspecialties [21–28]. Interpretation of cost-effectiveness remains limited by heterogeneity in procedures, platforms, and cost definitions across studies. Evidence regarding environmental impact is currently insufficient to support firm conclusions due to the scarcity of primary comparative data, underscoring the need for high-quality life-cycle assessments integrated with standardized economic analyses [29–32, 34]. Future research should adopt consistent cost frameworks and robust environmental methodologies to better inform sustainable and cost-effective instrument selection in minimally invasive surgery.
